# Serial ChIP as a tool to investigate the co-localization or exclusion of proteins on plant genes

**DOI:** 10.1186/1746-4811-4-25

**Published:** 2008-10-27

**Authors:** Zidian Xie, Erich Grotewold

**Affiliations:** 1 Department of Plant Cellular and Molecular Biology, The Ohio State University, Columbus, OH 43210, USA; 2 Plant Biotechnology Center, The Ohio State University, Columbus, OH 43210, USA

## Abstract

**Background:**

Establishing transcriptional regulatory networks that include protein-protein and protein-DNA interactions has become a key component to better understanding many fundamental biological processes. Although a variety of techniques are available to expose protein-protein and protein-DNA interactions, unequivocally establishing whether two proteins are targeted together to the same promoter or DNA molecule poses a very challenging endeavour. Yet, the recruitment of multiple regulatory proteins simultaneously to the same promoter provides the basis for combinatorial transcriptional regulation, central to the transcriptional regulatory network of eukaryotes. The serial ChIP (sChIP) technology was developed to fill this gap in our knowledge, and we illustrate here its application in plants.

**Results:**

Here we describe a modified sChIP protocol that provides robust and quantitative information on the co-association or exclusion of DNA-binding proteins on particular promoters. As a proof of principle, we investigated the association of histone H3 protein variants with modified tails (H3K9ac and H3K9me2) with *Arabidopsis *RNA polymerase II (RNPII) on the promoter of the constitutively expressed *actin *gene (At5g09810), and the trichome-expressed *GLABRA3 *(*GL3*) gene. As anticipated, our results show a strong positive correlation between H3K9ac and RNPII and a negative correlation between H3K9me2 and RNPII on the actin gene promoter. Our findings also establish a weak positive correlation between both H3K9ac and H3K9me2 and RNPII on the *GL3 *gene promoter, whose expression is restricted to a discrete number of cell types. We also describe mathematical tools that allow the easy interpretation of sChIP results.

**Conclusion:**

The sChIP method described here provides a reliable tool to determine whether the tethering of two proteins to the same DNA molecule is positively or negatively correlated. With the increasing need for establishing transcriptional regulatory networks, this modified sChIP method is anticipated to provide an excellent way to explore combinatorial gene regulation in eukaryotes.

## Background

In both eukaryotes and prokaryotes, regulation of gene expression is an essential process for most biological functions. Therefore, transcriptional regulation has been an active field of biological research over the past couple of decades [1]. The proteins involved in transcriptional regulation include the basic transcriptional apparatus and associated factors, such as the RNPII, the TATA-binding protein (TBP) and TBP-associated factors (TAFs), sequence-specific DNA-binding proteins and interacting cofactors, as well as histones and histone modifying proteins [2]. Proteins corresponding to several of these groups can form together a complex on the promoters of particular genes and facilitate the coordination between transcription initiation and elongation [3,4].

Establishing which proteins are located on any given gene promoter at a particular time will certainly contribute to understanding transcriptional regulation and the resulting gene regulatory networks. The genome-wide identification of transcription factor-DNA-interactions (TF-DNA) by high-throughput analyses such as chromatin immunoprecipitation (ChIP) coupled with the hybridization of promoter or tiling arrays (ChIP-chip) [5], as well as ChIP combined with massively parallel DNA sequencing (ChIP-Seq) [6] provide useful information on which regulators are targeted to which particular promoter sequences. However, such experiments fail to capture whether two TFs might be located together to the same promoter in the same cells simultaneously, or whether they bind to the same promoter but at different times or in different cells. High-throughput protein-protein interaction analyses, such as yeast two-hybrid [7] or TAP-tagging experiments [8], provide information on whether two regulatory proteins interact, yet they cannot determine whether the interaction is occurring on the DNA, for example, as part of the regulation of a common target, or before the two proteins are tethered to the DNA. Moreover, many of the proteins in a transcriptional complex may not necessarily physically interact, thus conventional methods to detect protein-protein interactions are not suited to determine the overall composition of the complex.

**Figure 1:**
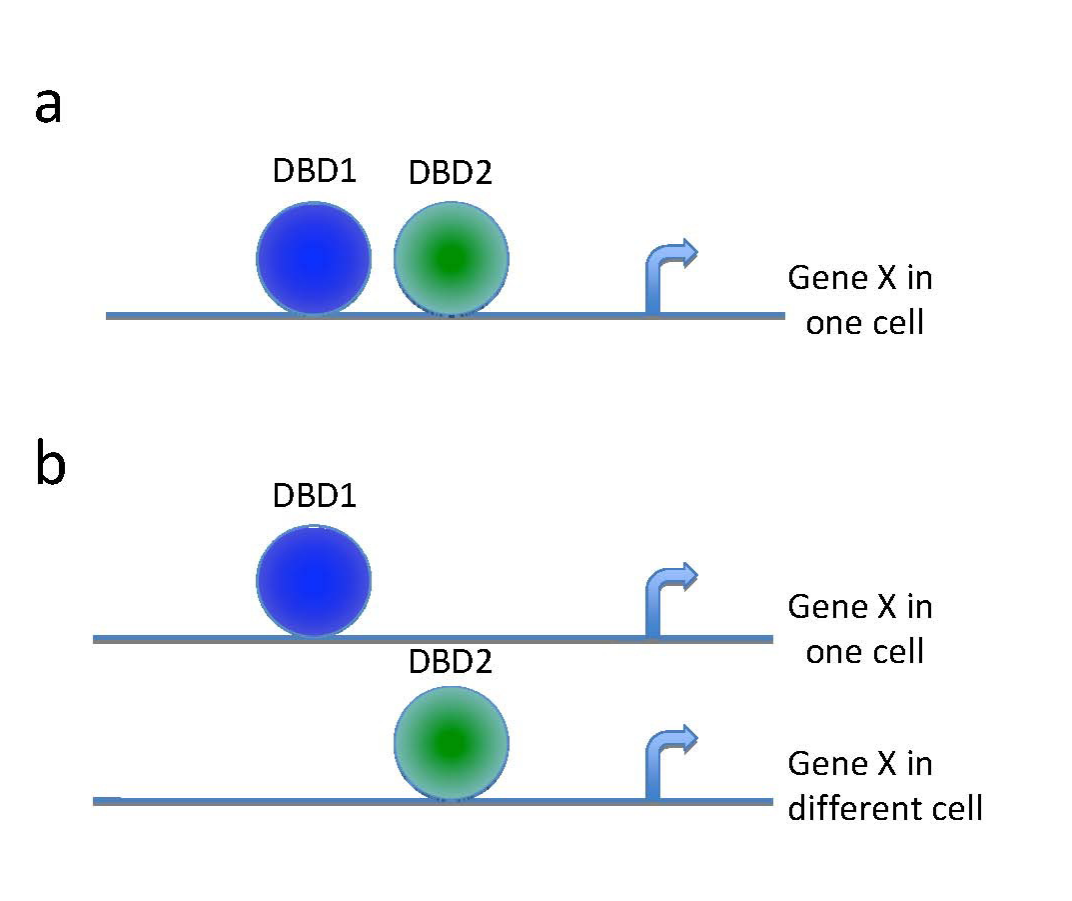
**Models for combinatorial transcription regulation between two proteins**. (a) The co-localization of two DNA-associated proteins DBD1 and DBD2 on the same gene X in the same cell. (b) The exclusion of two DNA-associated proteins DBD1 and DBD2 on the same gene X in the same cell. Both DBD1 and DBD2 can bind to the gene X, but in different cells.

The serial ChIP (sChIP) technique (a.k.a. re-ChIP and double ChIP) was recently developed to study the simultaneous association between two DNA-binding proteins on the same promoter, and was applied to yeast and mammalian system [9-15]. In brief, after the first ChIP is performed with antibodies for one of the proteins, a second ChIP is conducted on the DNA-protein complex from the first ChIP using an antibody that recognizes the second protein. As is the case for regular ChIP, the presence of a particular promoter fragment after the sChIP is verified by PCR. Thus, sChIP permits to establish the possible co-localization or exclusion of the two proteins on the same promoter (Fig. 1).

As part of the formation of the pre-initiation complex, RNPII is recruited to a region proximal to the transcription start site (TSS) of genes. Acetylated lysine 9 on histone H3 (H3K9ac) loosens the chromatin, which in turn facilitates the recruitment of TFs resulting in transcription [16]. In contrast, di-methylation of lysine 9 on histone H3 (H3K9me2) provides one of the markers associated with gene silencing and transcriptional repression [16]. Therefore, for genes transcribed at a particular time, H3K9ac and RNPII are expected to be present together (Fig. 1a), something that has been experimentally confirmed in human cells [17]. Similarly, for an expressed gene, the exclusion between RNPII and H3K9me2 is expected (Fig. 1b) [17].

Although sChIP has been used in a few instances to investigate whether two proteins co-localize to a particular gene promoter in yeast and animals, the technique has not been yet applied to plants. Most of these previous studies have focused on co-localization, yet exclusion of two proteins provides equally important information. Here, we describe a robust and reliable approach for sChIP in plants. By taking *Arabidopsis *RNPII and H3K9ac/H3K9me2 as examples, we show that sChIP is powerful in establishing the co-association (RNPII and H3K9ac) and exclusion (RNPII and H3K9me2) between two proteins on the same gene promoter. In addition, we show that for the *GL3 *gene promoter, intermediate levels of RNPII and H3K9ac/H3K9me2 association are observed, consistent with the cell-specific expression pattern (trichomes) of this gene in mature green plant tissues. Thus, this modified sChIP method provides a feasible and promising method to start uncovering the combinatorial transcriptional regulation code for plants.

## Results and discussion

### Modified sChIP

**Figure 2:**
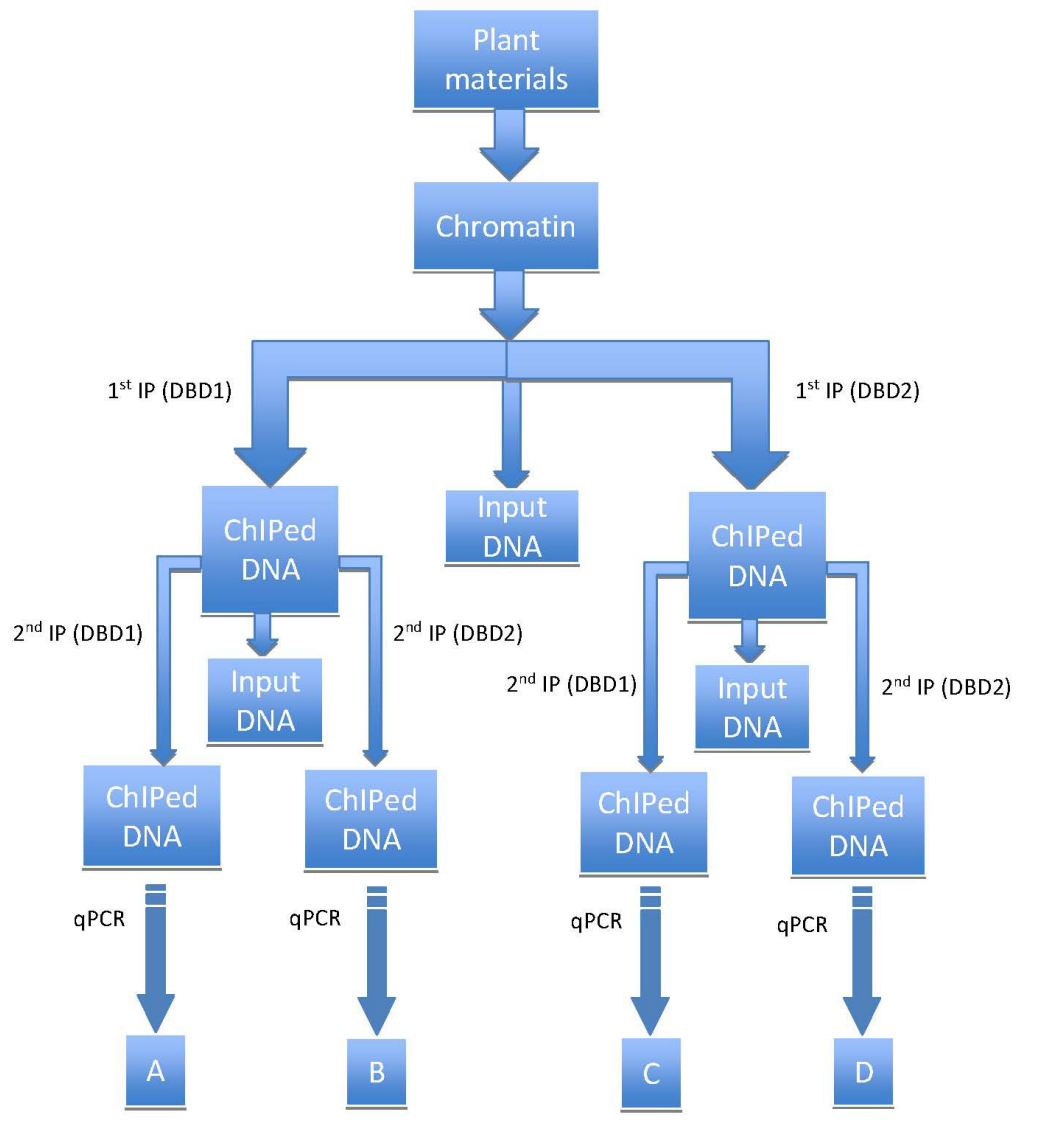
**Flow chart describing the modified sChIP procedure**. From *Arabidopsis *chromatin, the first ChIP was performed using antibodies that recognize either the DBD1 or DBD2 proteins, following the standard ChIP procedure. After washing and elution, the second ChIP was done using the antibody for either the DBD1 or DBD2 proteins on the products of the first ChIP. The amount of *actin *and *GLABROUS3 (GL3) *DNA present at each step (before and after ChIP) was evaluated using qPCR.

The standard ChIP and sChIP procedures are often associated with significant false positive and false negative results, which require extensive validation of identified interactions. In large part, this is due to variability in cross-linking and immunoprecipitation (IP), as well as by quality of the antibody used. As a first step in increasing the reliability of sChIP results, we developed a modified sChIP procedure (Fig. 2) based on standard ChIP [18,19] and sChIP procedures [13] and combining them with quantitative real-time PCR (qPCR) after each ChIP step, and careful data normalization and analysis.

### Theoretical calculations and predictions

#### Definitions and assumptions

**Figure 3:**
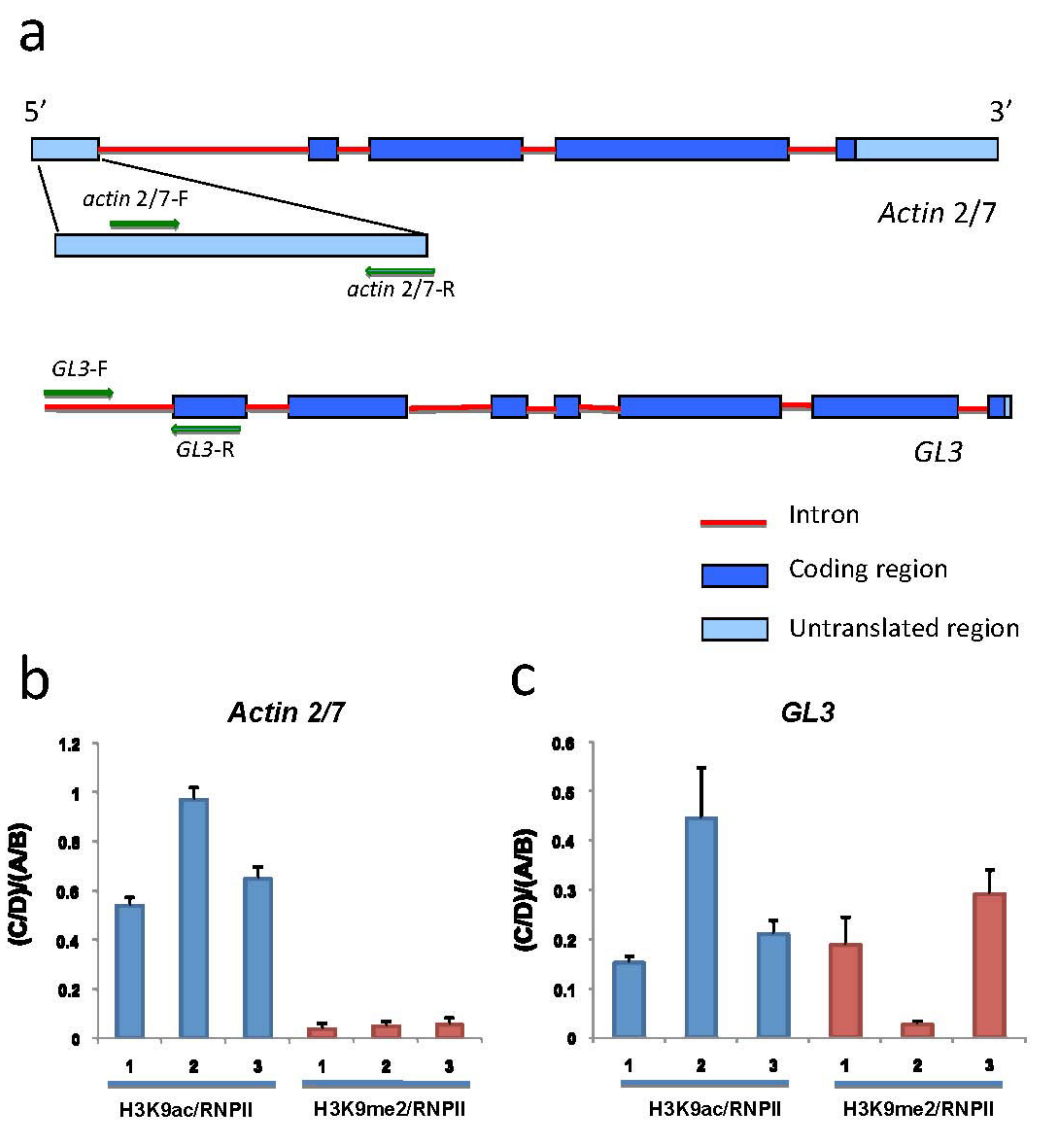
**sChIP data using antibodies for RNPII and histone proteins**. (a) Primer positions on the *actin *2/7 and *GL3 *genes. (b) sChIP data on *actin *2/7 using antibodies for H3K9ac and RNPII, and H3K9me2 and RNPII. (c) sChIP data on *GL3 *using antibodies for H3K9me2 and RNPII, and H3K9me2 and RNPII. The (C/D)/(A/B) ratios from three biological replicates were shown in (b) and (c). The error bar indicates the standard deviation of three technical replicates for the qPCR from each biological replicate.

1. "o" represents the percent of a promoter bound only by one DNA-associated protein (DBD1), "p" the percent of the same promoter bound by the second DNA-associated protein (DBD2), and "q" the percent of the promoter bound by both proteins.

2. We define ChIP efficiency as the percentage of the DNA that was ChIPed with a particular antibody to the same DNA bound by a particular protein in the corresponding input fraction, which can be the original chromatin for the first round of ChIP, or the DNA ChIPed by the first antibody in the second round of ChIP.

3. While it is tempting to assume that the ChIP efficiency is the same for the first and second IP using the same antibody and conditions, our experiments have shown that the ChIP efficiencies are very different (not shown). Thus, the ChIP efficiency for the DBD1 antibody at the first ChIP is "m1", and for the second ChIP is "m2". The ChIP efficiency for the DBD2 antibody is "n1", and "n2" for the second ChIP.

4. *Actin *(At5g09810) provides an example of a constitutively expressed gene in *Arabidopsis *[20], and primers in the 5' UTR of this gene (Fig. 3a) were used to evaluate the ChIP efficiency by qPCR (Materials and methods).

5. *GL3 *(At5g41315) provides an example of a gene that is expressed initially in the epidermis of young leaves and, as the leaf develops, becomes restricted to trichomes [21]. Primers for the promoter region of this gene were used (Fig. 3a) [18].

According to the scheme provided in Fig. 2, "A" denotes the ratio of sChIP final targeted DNA product using the DBD1 antibody followed by the DBD1 antibody to the starting targeted DNA; "B" denotes the sChIP using the DBD1 antibody followed by the DBD2 antibody; "C" denotes the sChIP using the DBD2 antibody followed by the DBD1 antibody; "D" denotes the sChIP using the DBD2 antibody followed by the DBD2 antibody.

Therefore, the ratio of final product "A or B or C or D" after sChIP to the total *actin *DNA in the sample can be calculated as follows:

A = o * m1 * m2

B = q * m1 * n2

C = q * n1 * m2

D = p * n1 * n2

Then,

C/D = q * n1 * m2/(p * n1 * n2) = q * m2/(p * n2)

A/B = o * m1 * m2/(q * m1 * n2) = o * m2/(q * n2)

Thus,

(C/D)/(A/B) = (q * q)/(o * p)

If two proteins are always present together at the same promoter molecule (perfect co-localization) (Fig. 1a), q = o = p. Therefore, C/D = A/B and (C/D)/(A/B) = 1.

If two proteins are never present together (exclusion) at the same promoter molecule (Fig. 1b), q = 0. Then, C/D will be significantly smaller than A/B, and (C/D)/(A/B) << 1.

### Serial ChIP applied to RNPII, H3K9ac and H3K9me2

To test this modified sChIP method, we used two well-characterized proteins: RNPII and different histone H3 variants (H3K9ac and H3K9me2) as examples. As a constitutive expressed gene, the *actin *2/7 gene (At5g09810) was used here as the target for both histone modifications and RNPII. As a trichome-specific gene, *GL3 *was used as non-constitutive expressed target. Using the antibodies for H3K9ac (corresponding for example to DBD1 in Fig. 2) and RNPII (corresponding for example to DBD2 in Fig. 2), sChIP was conducted on three biological replicates as described in Material and methods.

After quantified by qPCR, the "A", "B", "C", "D" values were calculated as the ratio of final ChIPed target DNA after sChIP to ten times of the same DNA in the corresponding original input fraction (1/10). The C/D and A/B ratios (Fig. 2) then were calculated. The need for performing qPCR rather than evaluating gene products by agarose electrophoresis is highlighted by the necessity to obtain quantitative estimates of the amount of DNA precipitated, and by the observation that the amount of DNA obtained from the second ChIP is very low and often cannot be visualized by conventional ethidium bromide staining after separation by agarose electrophoresis (not shown). Calculating the C/D and A/B ratios results in a normalization of the natural differences in efficiency that exist in ChIP experiments when using different antibodies. Despite the variation that exists between biological replicates (Fig. 3b), likely a consequence of different cross-linking efficiencies or chromatin quality, the A/B and C/D ratios are very close, which also can be visualized by the proximity of the (C/D)/(A/B) ratio to one (Fig. 3b). Indeed, there is no significant difference between A/B and C/D (*P *= 0.45, two-sided *t *test), suggesting that on the promoter of *actin *2/7, H3K9ac and RNPII co-exist, in agreement with the biological evidence [22]. Thus, if there is no significant difference between C/D and A/B, we can conclude that two proteins co-localize to that specific promoter at the same time.

To illustrate the exclusion of two proteins on the same gene, sChIP was conducted on three biological replicates using antibodies for H3K9me2 (corresponding for example to DBD1 in Fig. 2) and RNPII (corresponding for example to DBD2 in Fig. 2). The C/D and A/B ratios (Fig. 2) were computed based on qPCR data on ChIPed DNA and input DNA from each of the fractions (Fig. 2). The C/D ratio is much smaller than the A/B ratio in all three biological replicates, which results in the smaller (C/D)/(A/B) ratio (Fig. 3b). There is a significant difference between A/B and C/D (*P *= 0.0002, two-sided *t *test), indicating that on the promoter of *actin *2/7, RNPII and H3K9me2 localization are inversely correlated (called exclusion here), in agreement with the biological evidence that H3K9me2 marks transcriptional repression or silencing [21]. Thus, if C/D is significantly smaller than A/B, we can conclude that there is exclusion between two proteins on that specific promoter.

As a trichome-specific expressed gene, *GL3 *was used to illustrate the situation that histone H3 and RNPII are not always co-localized or excluded on the same promoter. As shown in Fig. 3c, there is a significant difference (*P *= 0.015, two-sided *t *test) between A/B and C/D using H3K9ac and RNPII, despite some variation between the three biological replicates. These results suggest that there is some level of exclusion between H3K9ac and RNPII on the *GL3 *gene promoter, which is consistent with the cell type restricted expression of *GL3*. Consistent with this, there is also a significant difference (*P *= 0.008, two-sided *t *test) between A/B and C/D using H3K9me2 and RNPII, indicating that there is some exclusion between H3K9me2 and RNPII. Interestingly, we noticed that there is negative correlation on (C/D)/(A/B) ratio between sChIP using H3K9ac/RNPII and H3K9me2/RNPII, especially between the first or third and the second biological replicate (Fig. 3c, replicate 2), which happens to be in agreement with the predicted biological functions of H3K9ac and H3K9me2.

## Conclusion

Using several examples, we have demonstrated that the modified sChIP method described here can be applied to detect the co-localization or exclusion of two proteins on the same DNA molecule in plants. Combined with qPCR and data normalization, this method overcomes the natural variation of ChIP efficiency between biological replicates and between different antibodies. As a proof of principle for plants, the sChIP protocol described here was proven to be highly reliable and sensitive, capturing the biological evidence that suggests co-localization of RNPII and H3K9ac, and the exclusion of RNPII and H3K9me2 on a constitutively expressed *actin *gene. In addition, and consistent with the cell-specific expression pattern, intermediate values were obtained for *GL3*. With the need for establishing the combinatorial transcriptional regulation network of plants, this modified sChIP method is likely to play a vital role in uncovering the co-association or exclusion of two proteins on the same DNA molecule.

## Methods

### Materials and reagents

Green tissues from ten days-old wild type *Arabidopsis *plants (*Columbia*) grown under continuous light at 22°C were used for sChIP experiments. The antibodies used for ChIP were as follows: αH3K9ac from Abcam (ab12178), αH3K9me2 from Abcam (ab7312), αRNPII CTD from Abcam (ab5131).

Buffer A: 0.4 M sucrose; 10 mM Tris pH 8.0; 1 mM EDTA; 1 mM PMSF (Freshly added from a 100 mM PMSF stock immediately before use); 1% formaldehyde

Lysis buffer: 50 mM HEPES pH 7.5; 150 mM NaCl; 1 mM EDTA; 1% Triton X-100; 0.1% sodium deoxycholate; 0.1% SDS; 10 mM Na-butyrate; 1 mM PMSF; 1X plant proteinase inhibitor cocktail (Sigma) (Freshly added from a 100× stock immediately before use)

LNDET: 0.25 M LiCl; 1% NP40; 1% sodium deoxycholate; 1 mM EDTA

TE buffer: 10 mM Tris-Cl, pH 7.5; 1 mM EDTA

Elution buffer: 1% SDS; 0.1 M NaHCO_3_; 0.25 mg/ml Proteinase K (Freshly added from a 10 mg/ml stock immediately before use)

Salmon sperm/protein A-agarose: from Upstate (16–157)

### ChIP Procedure

1. Immerse ~240 mg of *Arabidopsis *green tissue into buffer A in a 50 ml falcon tube and keep under vacuum (15–20 psi) for 20–40 min.

2. Add 2 M glycine to a final concentration of 0.1 M and continue vacuum for 10 min.

3. Wash the tissue with excess amount of distilled water and remove as much water as possible by kimwipe paper.

4. Grind tissue in liquid nitrogen and resuspend in 400 ml of lysis buffer.

5. Shear DNA by sonication to a range of 100–1,000 bp (~500 bp average) in an eppendorf tube. Using a Bioruptor (UCD-200TM, Diagenode Inc.), the sonication conditions are as follows: 30 seconds of sonication followed by 30 seconds of break at high power with 40 minutes in total.

6. Centrifuge at 10,000 × g for 10 min at 4°C.

7. Check the size of the DNA on a 1.5% agarose gel.

8. Pre-clear supernatant with 30 μl of salmon sperm/protein A-agarose slurry for rabbit polyclonal antibody for at least 60 min with rotation at 4°C.

9. After centrifugation at 3,000 rpm for 1 minute, transfer 100 μl of supernatant into three new 1.5 ml eppendorf tubes. Keep 10 μl as the input fraction and add the antibodies into three 100 μl fractions (1 μl H3K9me2 rabbit polyclonal antibody, or 1 μl H3K9ac rabbit polyclonal antibody or 1 μl RNP II rabbit polyclonal antibody).

10. Incubate overnight with rotation at 4°C.

11. Add 30 μl of salmon sperm/protein A-agarose slurry and continue incubation with rotation at 4°C for at least 2 hours.

12. Centrifuge at 750 × g (3000 rpm for microcentrifuge) for 1 min at 4°C.

13. Washes (at 4°C)

a) Add 0.5 ml of lysis buffer, invert 6 times, centrifuge at 750 × g for 1 min and discard supernatant.

b) Add 0.5 ml of lysis buffer, rotate for 5 min, centrifuge at 750 × g for 1 min and discard supernatant.

c) Add 0.5 ml of LNDET, invert 6 times, centrifuge at 750 × g for 1 min and discard supernatant.

d) Add 0.5 ml of LNDET, rotate for 5 min, centrifuge at 750 × g for 1 min and discard supernatant.

e) Add 0.5 ml of TE, invert 6 times, centrifuge at 750 × g for 1 min and discard supernatant.

f) Add 0.5 ml of TE, rotate for 5 min, centrifuge at 750 × g for 1 min and discard supernatant.

14. Add 40 μl of elution buffer and incubate at 65°C for 15 min.

15. Centrifuge at 750 × g for 1 min and transfer supernatant to new tube.

16. Repeat eluting steps. The final elution volume is 80 μl. In parallel, add 70 μl of elution buffer into 10 μl of input fraction for the 10% input control.

17. Incubate all samples overnight at 65°C.

18. Extract DNA by using PCR purification kit (QIAGEN). Elute in 30 μl of EB buffer (Tris-HCl, pH 8.5).

### sChIP

1. The immuno-complexes from the primary ChIP were resuspended, after washing, by incubating with 10 mM DTT at 37°C for 30 minutes.

2. After centrifugation at 3,000 rpm, the supernatant is diluted 1:50 in the dilution buffer (1% Triton X-100, 2 mM EDTA, 150 mM NaCl, 20 mM Tris-HCl, pH 8.1).

3. Using another antibody (Fig. 2), secondary immunoprecipitation was conducted in a similar manner as for the primary ChIP.

### qPCR and ChIP-PCR

Using SYBR-Green, qPCR experiments were conducted with the primers targeting to TSS site (Fig. 3a) of *actin *2/7 (At5g09810): *actin *2/7-F: 5'-CATGTACTCGTTTCGCTTTCC-3'; *actin *2/7-R:5'-AGCAGCAAAATCAAGCGAAC-3'. The primers for the promoter of *GL3 *(At5g41315): *GL3*-F: 5'-AAACGGCAACTGTTTCATCA-3'; *GL3*-R: 5'-TTCTGTTTTGTCCGGTAGCC-3'.

For ChIP-PCR, *actin *2/7-F and *actin *2/7-R were used. The PCR program was: 95°C for 5 min; 42 cycles of 95°C for 30 sec, 50°C for 30 sec, and 72°C for 1 min; 72°C for 10 min.

## Abbreviations

ChIP: chromatin immunoprecipitation; DBD1: DNA-associated protein 1; DBD2: DNA-associated protein 2; H3K9ac: histone H3 lysine 9 acetylation; H3K9me2: histone H3 lysine 9 di-methylation; qPCR: quantitative real-time PCR; RNPII: RNA polymerase II; sChIP: serial ChIP; TBP: TATA-binding protein; TAFs: TBP-associated factors; TF: transcription factor; *GL3*: *GLABRA3*; TAP-tagging: tandem affinity purification-tagging.

## Competing interests

The authors declare that they have no competing interests.

## Authors' contributions

ZX and EG designed the protocol described. ZX performed all the experiments and analyzed the data. ZX and EG co-wrote the manuscript. Both authors have read and approved the final manuscript.

## References

[B1] Wray GA, Hahn MW, Abouheif E, Balhoff JP, Pizer M, Rockman MV, Romano LA (2003). The evolution of transcriptional regulation in eukaryotes. Mol Biol Evol.

[B2] Carey M, Smale ST (1999). Transcriptional regulation in eukaryotes: concepts, strategies, and techniques.

[B3] Tuch BB, Li H, Johnson AD (2008). Evolution of eukaryotic transcription circuit. Science.

[B4] Tuch BB, Galgoczy DJ, Hernday AD, Li H, Johnson AD (2008). The evolution of combinatorial gene regulation in fungi. PLoS Biol.

[B5] Lee J, He K, Stole V, Lee H, Figueroa P, Gao Y, Tongprasit W, Zhao H, Lee I, Deng XW (2007). Analysis of transcription factor HY5 genomic binding sites revealed its hierarchical role in light regulation of development. Plant Cell.

[B6] Johnson DS, Mortazavi A, Myers RM, Wold B (2007). Genome-wide mapping of in vivo protein-DNA interactions. Science.

[B7] Ito T, Chiba T, Ozawa R, Yoshida M, Hattori M, Sakaki Y (2001). A comprehensive two-hybrid analysis to explore the yeast protein interactome. Proc Natl Acad Sci USA.

[B8] Gavin A, Bosche M, Krause R, Grandi P (2002). Functional organization of the yeast proteome by systematic analysis of protein complexes. Nature.

[B9] Kinoshita Y, Johnson EM (2004). Site-specific Loading of an MCM Protein Complex in a DNA Replication Initiation Zone Upstream of the c-MYC Gene in the HeLa Cell Cycle. J Biol Chem.

[B10] Lucey ML, Lucey MJ, Phoenix F, Lopez-Garcia J, Hart SM (2005). T:G mismatch-specific thymine-DNA glycosylase (TDG) as a coregulator of transcription interacts with SRC1 family members through a novel tyrosine repeat motif. Nucleic Acids Res.

[B11] Wang LH, Yang XY, Zhang X, An P, Kim HJ, Huang J, Clarke R, Osborne CK, Inman JK, Appella E, Farrar WL (2006). Disruption of estrogen receptor DNA-binding domain and related intramolecular communication restores tamoxifen sensitivity in resistant breast cancer. Cancer Cell.

[B12] Zhang H, Sun L, Liang J, Yu W, Zhang Y, Wang Y, Chen Y, Li R, Sun X, Shang Y (2006). The catalytic subunit of the proteasome is engaged in the entire process of estrogen receptor-regulated transcription. EMBO J.

[B13] Schnekenburger M, Talaska G, Puga A (2007). Chromium Cross-Links Histone Deacetylase 1- Methyltransferase 1 Complexes to Chromatin, Inhibiting Histone-Remodeling Marks Critical for Transcriptional Activation. Mol Cel Biol.

[B14] Chernukhin I, Shamsuddin S, Kang SY (2007). CTCF Interacts with and Recruits the Largest Subunit of RNA Polymerase II to CTCF Target Sites Genome-Wide. Mol Cel Biol.

[B15] Tworkowski KA, Chakraborty AA, Samuelson A, Segar YR, Narita M, Hannon GJ, Lowe SW, Tansey WP (2008). Adenovirus E1A targets p400 to induce the cellular oncoprotein Myc. Proc Natl Acad Sci USA.

[B16] Latham JA, Dent SYR (2007). Cross-regulation of histone modifications. Nat Struct Mol Biol.

[B17] Guenther MG, Levins SS, Boyer LA, Jaenisch R, Young RA (2007). A chromatin landmark and transcription initiation at most promoters in human cells. Cell.

[B18] Morohashi K, Zhao M, Yang M, Read B, Lloyd A, Lamb R, Grotewold E (2007). Participation of the *Arabidopsis *bHLH factor GL3 in trichome initiation regulatory events. Plant Physiol.

[B19] Morohashi K, Xie Z, Grotewold E (2009). Gene-specific and genome-wide ChIP approaches to study plant transcriptional networks. Methods Mol Biol.

[B20] An Y, McDowell JM, Huang S, McKinney EC, Chambliss S, Meagher RB (1996). Strong, constitutive expression of the *Arabidopsis *ACT2/ACT8 *actin *subclass in vegetative tissues. Plant J.

[B21] Zhao M, Morohashi K, Hatlestad G, Grotewold E, Lloyd A (2008). The TTG1-bHLH-MYB complex controls trichome cell fate and patterning through direct targeting of regulatory loci. Development.

[B22] Johnson LM, Cao X, Jacobsen SE (2002). Interplay between two epigenetic makers: DNA methylation and histone H3 lysine 9 methylation. Curr Biol.

